# Phylogenetic Analysis Reveals That ERVs "Die Young" but HERV-H Is Unusually Conserved

**DOI:** 10.1371/journal.pcbi.1004964

**Published:** 2016-06-13

**Authors:** Patrick Gemmell, Jotun Hein, Aris Katzourakis

**Affiliations:** 1 Department of Zoology, University of Oxford, Oxford, United Kingdom; 2 Department of Statistics, University of Oxford, Oxford, United Kingdom; Fred Hutchinson Cancer Research Center, UNITED STATES

## Abstract

About 8% of the human genome is made up of endogenous retroviruses (ERVs). Though most human endogenous retroviruses (HERVs) are thought to be irrelevant to our biology notable exceptions include members of the HERV-H family that are necessary for the correct functioning of stem cells. ERVs are commonly found in two forms, the full-length proviral form, and the more numerous solo-LTR form, thought to result from homologous recombination events. Here we introduce a phylogenetic framework to study ERV insertion and solo-LTR formation. We then apply the framework to site patterns sampled from a set of long alignments covering six primate genomes. Studying six categories of ERVs we quantitatively recapitulate patterns of insertional activity that are usually described in qualitative terms in the literature. A slowdown in most ERV groups is observed but we suggest that HERV-K activity may have increased in humans since they diverged from chimpanzees. We find that the rate of solo-LTR formation decreases rapidly as a function of ERV age and that an age dependent model of solo-LTR formation describes the history of ERVs more accurately than the commonly used exponential decay model. We also demonstrate that HERV-H loci are markedly less likely to form solo-LTRs than ERVs from other families. We conclude that the slower dynamics of HERV-H suggest a host role for the internal regions of these exapted elements and posit that in future it will be possible to use the relationship between full-length proviruses and solo-LTRs to help identify large scale co-options in distant vertebrate genomes.

## Author Summary

Animal genomes contain ancient pathogens known as endogenous retroviruses (ERVs). Though the widespread abundance of ERVs is due to their ability to self replicate, some ERVs are known to have become important to host processes including placentation, and in the case of HERV-H, the functioning of human stem cells. In our study we place the insertion and deletion activity of primate ERV families in direct quantitative comparison. In particular, we show that ERV deletion is an age dependent process, so that as an ERV ages it becomes less likely to be deleted at any given instant. We also find that ERVs from the HERV-H family are unusually slowly deleted, an interesting result that suggests that the exaptation of HERV-H may have involved internal regions of the virus and not just its terminal promoters. Assuming the behaviour of primate ERVs is not unusual, our study suggests that future bioinformatics screening for ERVs with slow deletion dynamics could help identify large-scale exaptations in distant species. Furthermore, as we demonstrate that ERVs are deleted rapidly, we think that such screening could be performed using ratios of conserved to deleted elements and could therefore be applied to single genomes.

## Introduction

By definition, endogenous retroviruses (ERVs) are the result of the Mendelian (vertical germ line) transmission of retroviruses from parent to progeny. Over many generations it is possible for an ERV to fix in a host population so that in humans, for example, as much as 8% of the genome is thought to be retrovirally derived [1]. Though the majority of fixed ERV loci are thought not to be under selection for function there are remarkable exceptions such as the *syncytins*[2] that are necessary for placentation, and members of the HERV-H family that are essential to stem cell identity in humans [3, 4].

Successful retroviral insertions (proviruses) are known to initially possess a common structure consisting of viral genes flanked by a pair of identical sequences known as long terminal repeats (LTRs). ERVs that retain this characteristic viral structure are commonly described as full-length ERVs. In addition to full-length ERVs, endogenized viruses are also found in a second, dramatically different form, referred to as a solo-LTR. A solo-LTR is a solitary LTR that is missing its associated partner LTR and adjacent proviral genes. Solo-LTRs are thought to be generated when paired LTRs undergo non-allelic homologous recombination which results in a deletion and an associated acentric fragment [5], a piece of chromosomal material lacking a centromere that is unlikely to persist across many cell divisions. Clearly, like other genomic DNA, both forms of ERVs are also subject to ordinary mutational processes so that over time they may become degraded or fragmented due to point mutations or indel events.

Studies have identified ERV activity dating back over millions of years and in many species e.g. [6–8]. When describing the replicational activity of viruses on evolutionary timescales many studies start by searching for full-length ERVs in a host genome. The resulting ERVs are then dated on an individual basis by comparing the divergence of paired LTRs that are assumed to have been identical at integration time. The aforementioned strategy is certainly reasonable but does have some limitations, including the fact that as the majority of ERVs are present in solo-LTR form [9] they do not contribute to analyses of replicational activity at all—whether or not this is problematic depends on the relationship between full-length proviruses and solo-LTRs.

In this study we analyze ERV insertion and solo-LTR formation in primates. Like other authors, we bring together insertion rates from several viral groups that are present in primates. Unlike other authors we do this by systematically sampling both full-length ERVs and solo-LTRs—in the same way for different ERV families and different host species—and relating these ERVs explicitly via a host genome alignment. By combining our sampling process with a host phylogeny we can then place insertion rates in quantitative comparison.

Our study is particularly concerned with the ratio of full-length ERVs to solo-LTRs. A study of the polymorphic HERV-K (HML2) loci in humans found that the majority of loci are represented by pre-integration sites or solo-LTRs [10]. This suggests that the solo-LTR formation process is very rapid. If this is true for most ERV families for most of the time then it is reasonable to treat a count of full-length ERVs as a constant fraction of a count of all ERVs, no matter the age of the infection. However, if the result does not generalize then such an assumption is not reasonable as the proportion of ERVs that are present in full-length versus solo-LTR form will vary given the age of an infection.

Within this paper we extend prior work by examining a wider variety of ERV families, by considering ERVs over a host phylogeny, and by introducing a likelihood framework that allows model comparison. Using this approach we confirm that there is an age dependent deletion process and are also able to demonstrate that HERV-H has unusual dynamics. Our results lead us to suggest that previously used models of deletion should be abandoned where possible. We also argue that a phylogenetic approach to characterizing ERV activity could help identify novel ERV exaptations in species distinct to those that we study here.

## Results

**Fig 1:**
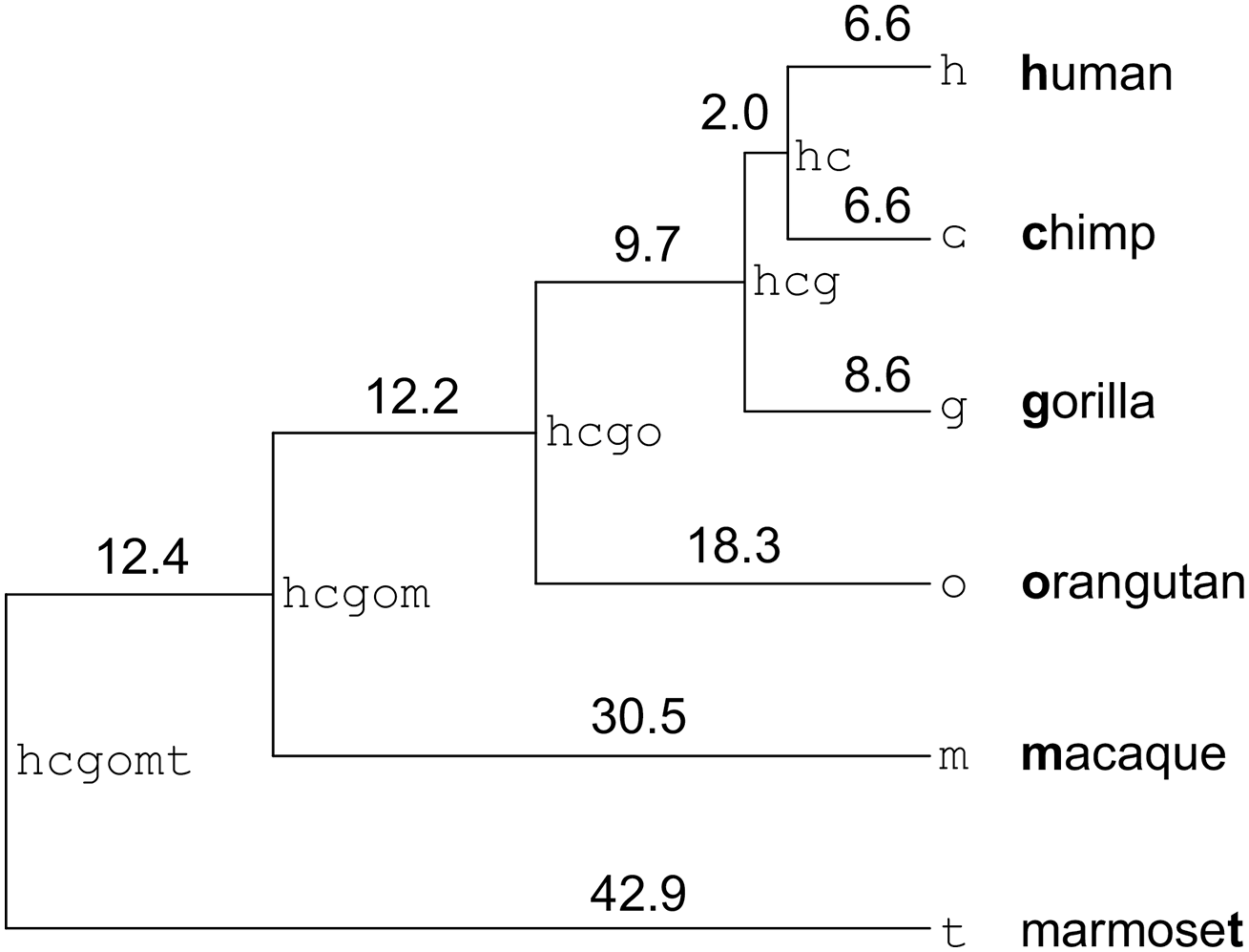
The primate host phylogeny used in this study. Nodes are labelled (fixed-width typeface) and branch lengths are given in millions of years (Myr) in accordance with [11].

We obtained site patterns describing ERV integrations and deletions that had occurred in the primate lineage (Fig 1) since the split between macaque and marmoset roughly 40 million years ago (Ma). The site patterns were obtained by relating post-processed RepeatMasker annotations to a six-way genome scale alignment of human, chimpanzee, gorilla, orangutan, macaque and marmoset sequence. These annotations were then converted to site patterns using a heuristic method. Our intention was to quantitatively describe the insertion rate of ERVs across branches of the primate phylogeny and also to investigate the process of ERV deletion that converts full-length ERVs into solo-LTRs.

Applying the methods sketched above, we identified 1,197 distinct insertion events that had occurred on the branch hcgom or later. These distinct insertions could be split naturally into groups based on the type of ERV they involved. We investigated the properties of insertions from the four largest families in our sample: ERV9 (245 insertions); HERV-K11 (197 insertions); HERV-H (116 insertions); and HERV-K (59 insertions). Based on a BLAST search against a library of 51 viral sequences, many ERVs from smaller families were assigned to group-I (131 insertions) or group-II (112 insertions). These patterns of insertion and deletion are presented in machine readable format in S1 Tabular data. Below we present the following results: (i) the insertion rate parameters obtained; (ii) the deletion rate parameters obtained under two different models of deletion; (iii) a comparison of the two competing deletion models; and (iv) the results of a simulation that tests the adequacy of the most appropriate deletion model.

**Fig 2:**
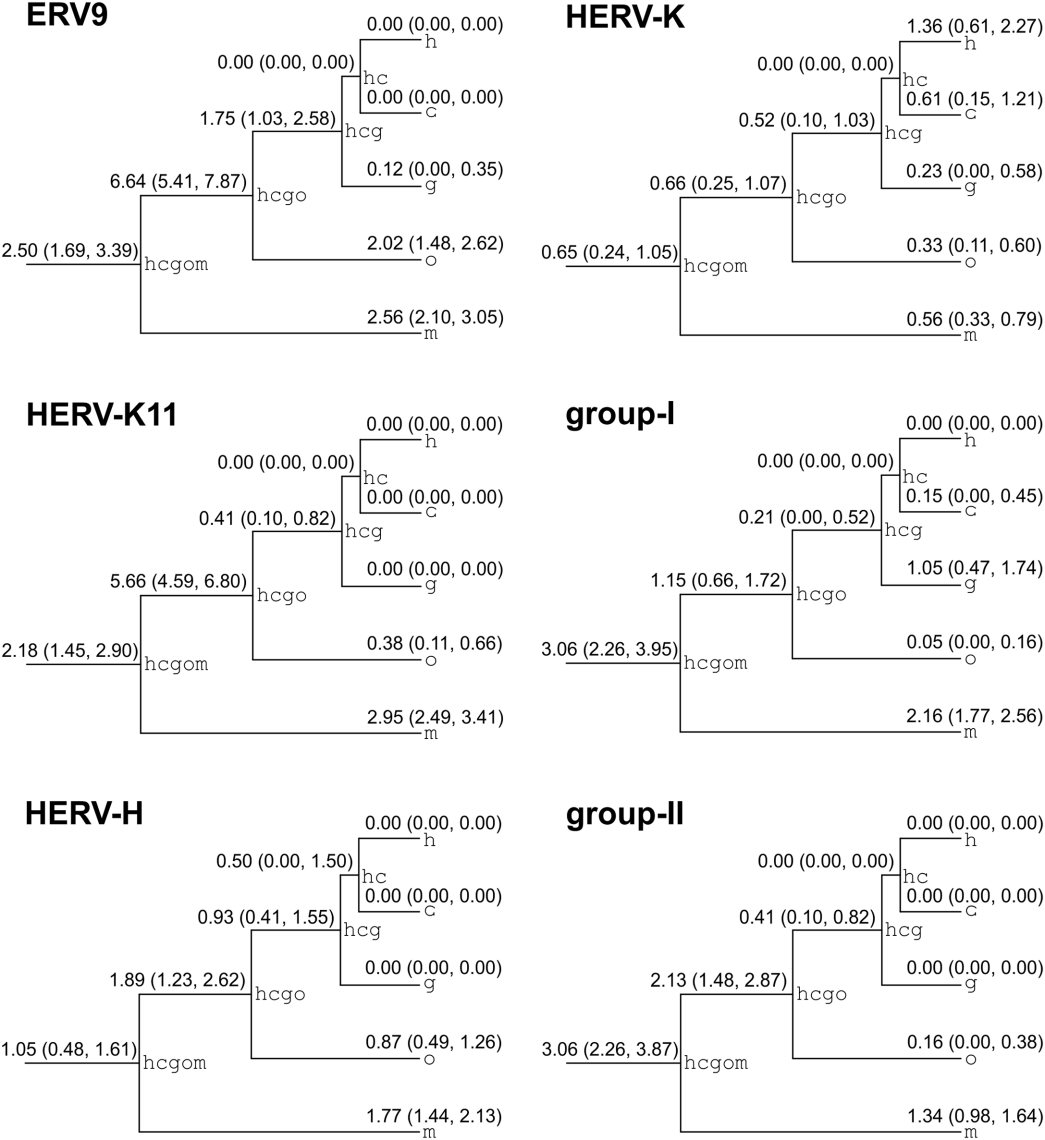
Branch and group specific maximum likelihood relative insertion rates in relative insertions per Myr. Bootstrap derived 95% confidence intervals are displayed in parentheses.

We applied our phylogenetic model (see Methods) to obtain the maximum likelihood relative insertion rates per million years (Myr) for the nine branches in our tree (Fig 2 and Table 1). These estimates of insertion activity are independent of the deletion model used. Our results are broadly compatible with descriptions contained in commonly cited studies on ERV dynamics e.g. [9, 12]. For example, the HERV-K insertion rates for branches h (1.36 relative insertions per Myr) and c (0.61 relative insertions per Myr) capture the commonly reported fact that HERV-K has been recently active in both human and chimp and also that the activity in human specific ancestors appears to have been at least 50% greater than the activity in chimp specific ancestors. In general, more detailed comparison is difficult as individual studies vary considerably in methodology and reporting style, a state of affairs that partially motivated us to perform the analysis reported in this paper.

**Table 1:** Branch and group specific maximum likelihood relative insertion rates in relative insertions per Myr. The absolute insertion counts giving rise to the rates are shown in parentheses and the corresponding site patterns are provided in S1 Tabular data.

	ERV9	HERV-K11	HERV-H	HERV-K	group-I	group-II
h	0.00 (0)	0.00 (0)	0.00 (0)	1.36 (9)	0.00 (0)	0.00 (0)
c	0.00 (0)	0.00 (0)	0.00 (0)	0.61 (4)	0.15 (1)	0.00 (0)
g	0.12 (1)	0.00 (0)	0.00 (0)	0.23 (2)	1.05 (9)	0.00 (0)
o	2.02 (37)	0.38 (7)	0.87 (16)	0.33 (6)	0.05 (1)	0.16 (3)
m	2.56 (78)	2.95 (90)	1.77 (54)	0.56 (17)	2.16 (66)	1.34 (41)
hc	0.00 (0)	0.00 (0)	0.50 (1)	0.00 (0)	0.00 (0)	0.00 (0)
hcg	1.75 (17)	0.41 (4)	0.93 (9)	0.52 (5)	0.21 (2)	0.41 (4)
hcgo	6.64 (81)	5.66 (69)	1.89 (23)	0.66 (8)	1.15 (14)	2.13 (26)
hcgom	2.50 (31)	2.18 (27)	1.05 (13)	0.65 (8)	3.06 (38)	3.06 (38)

Node names are as per Fig 1.

As well as insertion, we are also interested in the process by which ERVs are deleted. The simplest conceivable model of a deletion process is one which has a constant hazard over time, i.e. a process under which the probability of deletion in an infinitesimally small period of time is constant. The unique process with this property is an exponential decay process. Under the exponential model, the probability of deletion of an ERV is independent of its age. Such a model is appropriate if the probability of deletion of an ERV is small and fairly constant across generations, and if the probability of deletion of an ERV has nothing to do with the process by which an ERV ages. For each of the six groups of ERV we obtained a maximum likelihood estimate of exponential rate parameter *ψ*_*e*_ (Table 2).

**Table 2:** Group specific log likelihoods and corresponding maximum likelihood estimates of parameter values under exponential and Weibull deletion processes. Parameters were constrained to the interval 10^−6^ to 10^2^.

	*ℓ*_*e*_	*ℓ*_*w*_	*ψ*_*e*_	*ψ*_*w*_^−1^	*ω*	2Δ*ℓ*
ERV9	−158.54	−107.55	0.14	> 100.00	< 0.14	101.98
HERV-K11	−144.83	−74.68	0.11	> 100.00	< 0.12	140.31
HERV-H	−140.88	−121.79	0.05	= 0.09	= 0.18	38.18
HERV-K	−58.46	−32.33	0.16	> 100.00	< 0.13	52.27
group-I	−109.17	−47.54	0.10	> 100.00	< 0.13	123.26
group-II	−89.25	−50.62	0.10	> 100.00	< 0.12	77.27

Under the exponential model, full-length elements from the HERV-K family would be deleted most quickly, with full-length loci having an average pre-deletion lifetime of approximately 6.25 Myr. Under the same model, the most long-lived group would be HERV-H, for which the average full-length lifetime would be roughly 20 Myr. At an age of 400,000 years (the expected fixation time of a neutral ERV given an effective population size of 10,000 and a generation time of 10 years), the exponential model predicts that 94–98% of ERVs would retain their full-length form. By an age of 25 Myr, a time period comparable to the scope of our phylogeny, the exponential model predicts that only 2% of HERV-K insertions would remain in full-length form while a much larger 29% of HERV-H insertions would.

**Fig 3:**
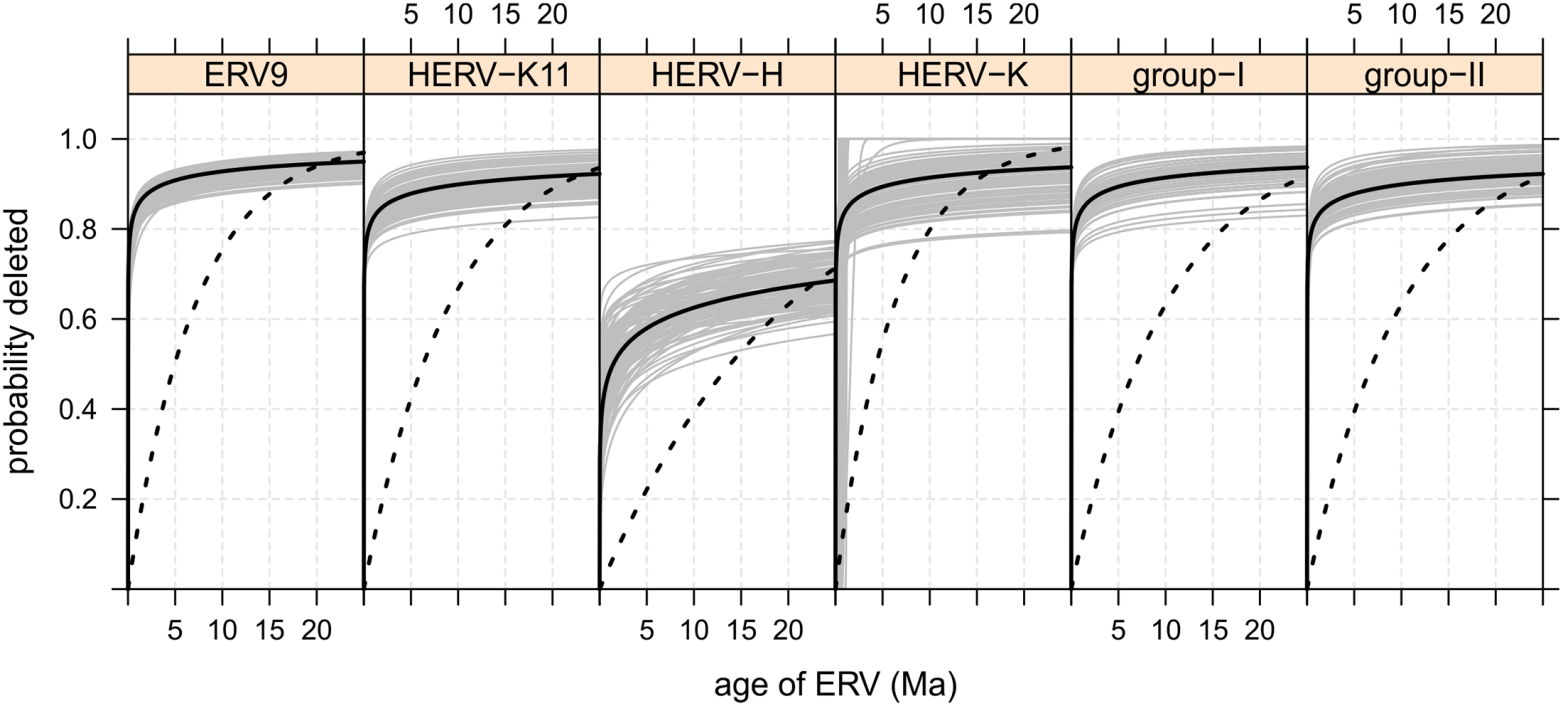
Group specific CDFs describing the probability of deletion of an ERV given its age. The CDFs are derived using maximum likelihood parameter estimates under a Weibull model (thick black line), from bootstrap resampled site patterns under a Weibull model (thin grey lines), and from maximum likelihood estimates under an exponential model (black dotted lines).

Beyond the simplest possible deletion scenario, we are also interested in the hypothesis that the formation of solo-LTRs is governed by a process that depends on the age of an insertion i.e. a process with a variable hazard. For example, it may be that as ERVs age, substitutions and gene conversion introduce differences between paired LTRs that substantially reduce their chance of producing solo-LTRs. A process with a hazard rate that changes with time is often modeled using a Weibull distribution. Under this process the rate of deletion is proportional to a power of time so that the probability of the removal of a full-length ERV can decrease with age, given a shape parameter *ω* < 1, or increase with age, given a shape parameter *ω* > 1. A shape parameter of *ω* = 1 implies an exponential decay process so that the exponential model is a nested submodel of the Weibull model. For each of the six groups of ERVs we obtained a maximum likelihood estimate of scale parameter *ψ*_*w*_ and shape parameter *ω* (Fig 3, Table 2).

Under a Weibull model, we find that at an early age it is again ERVs from the HERV-H family that are most likely to remain in full-length form. We find that at an age of 400,000 years 58% of HERV-H would remain in full-length form whereas only 19–21% of the other five groups would. These predictions differ by 40–76 percentage points from those of an exponential model. At the longer time scale, maximum likelihood parameter estimation suggests that at an age of 25 Myr we expect 31% of HERV-H to remain in full-length form while we expect less of ERV-9 (5%), HERV-K11 (8%), HERV-K (6%), group-I (6%) or group-II (8%) to do so. Summarizing a Weibull deletion process requires considering the role of the shape parameter. The maximum likelihood estimate of shape parameter *ω* is less than 1 for all six groups of ERV, and therefore suggests the rate of ERV deletion does decrease monotonically with time. Bootstrap replicates suggest this is unambiguously true for all families apart from HERV-K, for which 12% of bootstrap replicates identify a shape parameter >1. This implies that there is a degree of uncertainty over whether HERV-K has qualitatively different dynamics than the other ERV groups, with these exceptional bootstrap estimates suggesting peak deletion rates occur at ages of up to just over 4 Myr.

We have described the results of fitting two competing models, formalized as the null hypothesis *H*_*e*_ that the deletion process is an exponential decay, and as the alternative hypothesis *H*_*w*_ that the deletion process is age dependent. To decide which of the models is more appropriate we performed a likelihood ratio test. As *H*_*e*_ is nested within *H*_*w*_, which has one additional parameter, we compare 2Δ*ℓ* = 2(*ℓ*_*w*_−*ℓ*_*e*_) with a *χ*^2^ cutoff of 10.83. We find that 2Δ*ℓ* implies that the Weibull age specific model is clearly more appropriate for all six groups of ERV (*p* < 0.001).

Our likelihood values show that a Weibull model is a much better description of the ERV deletion process than an exponential model. However, likelihood ratio tests do not provide an assessment of the adequacy of the Weibull model itself. For this reason we conducted a simulation to see whether the Weibull model could explain the empirical site patterns we observed for each of the six ERV groups. Our simulation proceeded as follows. For each of the six ERV groups, we generated 10,000 insertions on branch hcgom, the deepest branch in our phylogeny. We then simulated the history of these insertions according to the Weibull model operating under group specific maximum likelihood estimates of *ψ*_*w*_ and *ω*. This generated the group specific frequency distribution of site patterns at the tips of the tree. We performed a goodness of fit test comparing the distribution of empirical site patterns with the frequencies obtained via simulation. We found no statistical evidence that the distribution of observed site patterns differed from those expected under the Weibull model for any of the six groups (Table 3). This suggests a Weibull model is an adequate one.

**Table 3:** Group specific two-sided goodness of fit tests.

	n	*H*_*e*_	*H*_*w*_
ERV9	31	*p* < 0.01	*p* = 0.48
HERV-K11	27	*p* < 0.01	*p* = 0.37
HERV-H	13	*p* = 0.60	*p* = 0.28
HERV-K	8	*p* < 0.01	*p* = 0.53
group-I	38	*p* < 0.01	*p* = 0.25
group-II	38	*p* < 0.01	*p* = 0.56

Fisher exact tests show that the distribution of observed site patterns is not significantly different from those expected under Weibull deletion model *H*_*w*_. This is not true in general for the exponential decay model *H*_*e*_.

## Discussion

In this paper we sample ERV site patterns from the primates and present a phylogenetic model (see Methods) which we show captures the deletion process of ERVs in a way that is superior to existing descriptions. Applying this model to data on six large groupings of ERVs we find that HERV-H is the most slowly deleted group of ERVs across the primate phylogeny and that, with the potential exception of HERV-K, ERVs appear to “die young.” Below we discuss the biological implications of our findings, the limits of our approach, and why we think our approach can help identify exapted ERV families in other lineages of vertebrates.

Previous studies of ERV activity (i.e. insertions) have often proceeded by enumerating the full-length ERVs, perhaps of a specific type, from one host species. Meta-studies will then collate the results of primary studies and attempt a synthesis of their contents e.g [9, 12]. Meta studies face the difficult problem of relating various sampling (search) methodologies. They also face the impossible problem of relating counts of full-length ERVs between species when the overlap between counts is unknown. Here we think our approach is helpful. Consider Fig 2, where we provide estimates that allow one to answer quantitative questions about the insertional activity of ERVs from different families and species. Assuming our sampling of viruses has been effective, we can be confident that both group-I and group-II ERVs became less active after the split of the macaque lineage from the ape lineage, but also that ERV9 and HERV-K11 were more active in the ape lineage after this split than before. As we measure insertions using solo-LTRs as well as full-length ERVs, we also suggest that, contrary to a previous conclusion [13], an apparent speedup in HERV-K(HML2) is not an artefact of considering only full-length ERVs. Therefore we think our results complement existing research. We also think that similar approaches will become more useful as newly sequenced genomes give better resolution within phylogenies and allow for improved genome scale alignments.

The topic of solo-LTR formation has been less widely studied in the past, perhaps because it is assumed to be unimportant to host phenotype, or perhaps because an adequate treatment requires phylogenetic data. What is true is that when a deletion process is explicitly mentioned it is often assumed to be exponential or constant over time e.g. [14] or [15]. Our results show that estimates produced assuming a constant deletion model differ dramatically from those produced using an age dependent model. We also show that the assumption of a pan-lineage constant deletion process is clearly inappropriate for five of the six groups of ERVs we examined in primates. We have no *a priori* reason to consider other deletion scenarios (e.g. branch specific deletion) and conclude that, pending further research, an exponential model is inappropriate more generally.

The question of whether ERV deletion rates vary with age was previously addressed by Belshaw et al. [16] who reported that the deletion rate for recent integrations was 200 fold higher than for integrations that occurred over 6 Ma. This effect was ascribed to mutational divergence between LTRs reducing homologous recombination although background genomic recombination might also play a role [17]. The result of Belshaw et al. [16] is implicitly conditional on loci being retained in full-length form in at least one of human or chimpanzee and, to our knowledge, while commonly cited, has not been followed up elsewhere. Our results generalize the qualitative conclusions of Belshaw et al. [16] to the primate phylogeny and to a variety of ERV families. This is important because the previous results rely exclusively on elements from the HERV-K category which are unusual for two reasons. First, the HERV-K family of ERVs have recently been insertionally active. Second, some recent HERV-K insertions have been shown to have biochemical effects, including virion formation, during early stages of human development [18]. In addition, our results surpass previous ones as we provide a description (a parameterized Weibull model) that gives an indication of the expected survival function of an ERV at various points in its lifetime.

Given that divergence between LTRs is hypothesised to be responsible for an age dependent deletion process [16], it is interesting to consider mutations into the LTRs of unfixed ERVs. Experiments by Datta et al. [19] show that a single nucleotide difference between two 350 base pair (bp) substrates can lower recombination 3-fold in yeast. Opperman et al. [20] find a 4-fold reduction in recombination when using 618 bp substrates in plants. Both studies find that additional mutations have relatively little effect. Bearing these data in mind, simulation using a Wright-Fisher population of 10,000 individuals shows that a (conservative) mutation rate of 10^−8^ per site per generation will introduce a difference into a neutrally segregating pair of 1,000 bp long LTRs by generation 215 on average. The expected frequency of a full-length ERV at the time a difference is introduced is less than 1%, and an ERV can be expected to have a complementary mutant by the time it has reached a frequency of 4% at most. Further, if we assume deletion does not occur, an ERV with mutation free LTRs will fix on only 33% of occasions and an ERV’s LTRs will contain 1 (24% of occasions), 2 (15% of occasions) or 3 or more mutations (28% of occasions) upon fixation otherwise. Thus, simulation suggests that if, as experimental evidence suggests, divergence plays a substantial role in reducing recombinational deletion then deletion should usually occur quickly. If this were not the case then most ERVs that fixed would already contain mutations that hindered deletion, an outcome that would seem to contradict the empirical fact that most ERVs are found in solo-LTR form. In other words, simulation appears to theoretically support our findings.

Not all ERVs are deleted equally quickly. Among the groups of ERVs we examined we found that HERV-H were unusually slowly deleted. This is interesting for several reasons. HERV-H is notable as a family because there is very strong evidence that some HERV-H loci are essential for the maintenance of stem cell identity in humans [3, 4]: half of the full-length HERV-H loci in the human genome are bound by pluripotency associated transcription factors NANOG, OCT5 and LBP9, causing them to produce chimeric stem cell specific transcripts and long non-coding RNAs. It has also been demonstrated that highly transcribed HERV-H loci have diverged faster since the chimp-human split at the nucleotide level than other ERVs and other repetitive DNA [21]. Here our results show that HERV-H loci are more likely to be preserved in a full-length state than ERVs from any of the other five groups we examined. As well as being consistent with the aforementioned mutational divergence hypothesis, this finding may also suggest that full-length HERV-H are useful to the host in a way that solo-LTRs are not. Both solo-LTRs or LTRs that are part of full-length proviruses can act as promoters; we think an implication of our findings is that HERV-H exaptation may involve more than the provision of an LTR promoter and that additional study of the internal regions of HERV-H loci is necessary.

Of course, although we favour the idea that full-length HERV-H is selected for—in the sense that selection may prefer full-length ERVs to solo-LTRs in some instances—there are certainly other hypotheses that could be put forward. Indeed, while we have previously argued that the rapid divergence of what appear to be functional ERVs is evidence of directional selection [21], one could also argue that there might be some other reason that HERV-H is fast evolving. Additionally, it is also possible that full-length HERV-H might be particularly benign, so that for some reason it is easier for full-length HERV-H to fix than for full-length ERVs of other kinds. This idea has some biological justification because it is known that HERV-H envelope genes—involved in cell entry and immunosuppression in exogenous retroviruses—are generally highly degraded [22, 23]. This is not necessarily the case for other full-length ERVs and may have helped full-length HERV-H spread [24]. Alternatively, the unknown number of HERV-H elements that have been exapted may have caused primates to be more tolerant of HERV-H expression in general, making it easier for non-exapted full-length elements to fix. These discussions highlight complex issues, though if one were able to definitively identify those particular HERV-H that had been exapted then some progress could be made. For example, using independent data to classify ERVs as exapted or otherwise, one could determine whether full-length HERV-H are deleted at the same rate as other ERV groups in the case that they are not exapted. In such a scenario, the ERV deletion parameters we show in Fig 3 would probably turn out to be an underestimate of deletion probabilities for non-exapted elements and an overestimate for the others.

It is important to discuss the limits of our model. While phylogenetic assignment of insertions to branches can potentially be more precise than assignments based on LTR dating, such assignment is limited by the resolution of the phylogeny used. This is clear from Fig 2. The branches leading to human and chimpanzee generally reflect a decrease in ERV activity while the branch leading to macaque can only reflect the average activity over a 30 Myr period. Although the average is similar to the average over internal branches, it is probable that the majority of ERV insertions actually occurred closer to the origin of the macaque lineage than to the present day. It is therefore likely that data from the macaque branch will bias deletion rates in the direction of overestimation. Additionally, our approach assumes that ERVs arrive in full-length form. This assumption was necessary, but it is reasonable to point out that some site patterns—those with xs at every tip—describe ERV integrations that might never have been passed vertically from generation to generation in a full-length form. Empirical studies of active ERV families such as KoRV [25] can potentially tell us what proportion of loci can be expected to endogenize in solo-LTR form. Finally, our model is limited by the availability of full-length insertion data. Branches for which all insertions have resulted in solo-LTRs provide no upper bounds on the rate of deletion. For this reason we performed parameter inference on the interval 10^−6^ to 10^2^, a range broadly compatible with the resolution of our phylogeny. Larger datasets or improved sampling of site patterns can resolve this problem.

Limitations notwithstanding, we hope to show that ERV activity is well described using phylogenetic techniques that avoid LTR dating, and that previous arguments that younger ERVs are more quickly deleted than older ones can be correctly formalized. We also suggest that our finding that HERV-H is deleted slowly across the primate phylogeny supports a long term biological role for some full-length members of the family. Finally, given HERV-H loci seem to have been subject to slow deletion, fast divergence, and are sometimes actively transcribed, we suggest that the *in silico* identification of ERV families with similar dynamics in other species might be expected to highlight other large scale co-option events. Indeed, a consequence of generalizing the rapid and age dependent nature of solo-LTR formation is the knowledge that such exaptations may be identifiable by locating families with unusually high full-length to solo-LTR ratios within a single genome. From this perspective HERV-H is an outlier in our dataset (S1 Tabular data: Summary of ratios) and others [9]. Such an approach would be reasonable when comparing families with sufficiently similar past activity and when the insertions under consideration are old enough that one would expect rapid deletion to have converted the majority of non-exapted elements into solo-LTRs.

### Conclusions

We sample site patterns describing the state of endogenous retroviruses (ERVs) in six primate species and analyze the process of insertion and deletion. Using a phylogenetic framework, we place the activity of six categories of ERV in direct quantitative comparison for the first time. We find that ERVs “die young” but that HERV-H loci are markedly more long-lived than ERV9, HERV-K11, HERV-K, or other class-I and class-II loci. To us, the lower probability of solo-LTR formation for HERV-H loci suggests a long-term host role for the internal regions of exapted elements.

We show that an age dependent Weibull model is sufficient to describe the ERV deletion process and that the use of an exponential decay process is less accurate. As the observation that solo-LTR formation occurs rapidly can be phylogenetically formalized, and generalizes to the majority of ERVs in primate genomes, we propose that the ratio of full-length to solo-LTRs may be used to help identify exapted ERV families in other vertebrate lineages.

## Materials and Methods

In overview, our method was to collect a sample of ERV site patterns from a variety of primates, and to use those patterns, in combination with a host phylogeny, to find the maximum likelihood parameter values for two variations of insertion and deletion processes. This allowed us to decide which model process fit our data best. Below we first describe the process of sampling site patterns and then introduce our phylogenetic techniques.

### Alignment and repeat annotation

We obtained the six-way Enredo-Pecan-Ortheus (EPO) whole genome multiple alignment of primate species that forms part of Ensembl Release 71 [26]. The six species included in the alignment are: human (*Homo sapiens*), chimp (*Pan troglodytes*), gorilla (*Gorilla gorilla*), orangutan (*Pongo abelii*), macaque (*Macaca mulatta*) and marmoset (*Callithrix jacchus*).

The EPO dataset contains 7,224 individual alignments containing exactly one sequence for each of the six species. These specific alignments comprise approximately 32% of all alignments in the dataset, which also covers duplicate regions and regions that are present or alignable in only a subset of the six primates. The 7,224 alignments were usually of the order of 10^5^ to 10^6^ columns in length and had a median LTR content of 8%. To identify LTR retroelements we ran RepeatMasker 3.3 [27] on each ungapped sequence from each individual alignment using the -species mammal -no_is -pa 4 -q -nolow -norna options.

The repeats identified by RepeatMasker are often fragmented so that an LTR element originating from a single insertion event is referenced using several distinct annotations. For this reason we applied REannotate 26.11.2007 [28] using options -c -n -f to our RepeatMasker results. This resulted in the identification of complete ERVs, truncated ERVs, and solo-LTRs, entities corresponding to distinct insertional events as defined by [28]. The application of REannotate also conveniently mapped synonymous RepeatMasker identifiers to an appropriate canonical identifier e.g. identifiers HERVH, LTR7, LTR7Y, RTVL-H, RTVL-H2 and RGH were all mapped to identifier HERV-H.

The result of the above repeat masking and annotation processes was data giving the location, repeat type, and structural status of LTR elements in ungapped coordinates. These ungapped coordinates could be mapped back to the appropriate locations in the original EPO multiple alignment files.

To check that REannotate had assigned the correct identifiers to ERVs we performed a BLAST [29] alignment of a representative sequence underlying each repeat locus against a library of 51 viral sequences drawn from [30] and [31]. For any given alignment locus, a full-length representative sequence was preferred to a solo-LTR where available. Representative sequences were drawn from human or the closest primate to human in preference to those that were more distant. For each repeat assigned to families ERV9, HERV-K11, HERV-H and HERV-K, we also submitted the sequences to Dfam [31], and as BLAST queries to NCBI to examine their structure in detail. This resulted in the removal of 8 SVAs that would have otherwise been erroneously included in our study.

### Construction of site patterns: overview and assumptions

#### From alignments to site patterns

To obtain site patterns from annotated alignments we first partitioned them into regions of contiguous columns containing identical features in any given row (Fig A in S1 Illustrated methods). Because identical columns are completely redundant we collapsed these regions into vectors which we call pre-patterns (Fig B in S1 Illustrated methods). We then scanned these pre-patterns for changes in the relationship between sequences, allowing us to detect insertions and deletions which we described using site patterns (Figs C and D in S1 Illustrated methods). Our assumption was that labelling columnar regions on a per-species basis would provide enough information to identify the state of orthologous integration sites (full-length ERV, solo-LTR, pre-integration site) in the six primates. We were interested in gross differences between sequences comprising an alignment—so that we could detect insertions and deletions—but we were not interested in the fine details, and for this reason we ignored pre-patterns describing regions of less than 50 bp in length. We also assumed we could ignore nested insertions without biasing our results.

#### Post-processing site patterns

Our experience examining potential site patterns suggested that occasionally ERVs would be labelled by REannotate in one species but not in another. To deal with this we assumed that sequence that was unannotated in one species could inherit the annotation of a sequence to which it was completely aligned. Furthermore, we found that a heuristic that relabelled solo-LTRs as full-length ERVs if they were 85% the length of the corresponding full-length element was appropriate. We ignored patterns that were less than 250 bp long in total as manual inspection suggested these regions could not necessarily be reliably called.

Sometimes we found that the region of a sequence in which an insertion might otherwise have been observed in was missing from an alignment. To deal with this we ignored site patterns that were not flanked with at least 100 bp of host DNA. This allowed us to assume that pre-integration sites had been aligned and that gaps genuinely indicated the absence of an insertion in a particular species.

#### Other assumptions

Finally, we remark that our phylogenetic model cannot deal with incomplete lineage sorting or segmental duplications and deletions. For this reason we restricted our analyses to alignments containing exactly six sequences and also ignored site patterns that may indicate incomplete lineage sorting. Our assumption was that ignoring these alignments and patterns would not systematically bias our results.

### Construction of site patterns: Detailed description

Consider a six-way alignment of length *m*. We form a corresponding 6 by *m* classification matrix *A* = {*a*_*i*,*j*_} in order to combine the information output from the REannotate program with information contained in the alignment. Each entry *a*_*i*,*j*_ is conceptually of one of the following kinds: an unannotated nucleotide coded as d; an unannotated gap coded as g; the *i*th solo-LTR having identifier *id* coded as s- *i* - *id*; or the *i*th partial or full-length ERV having identifier *id* and coded as c- *i* - *id*. For example, positions annotated as belonging to the fourth full-length HERV-H in an alignment would be given the classification c-HERV-H-4.

#### Converting an annotated alignment into pre-patterns

We partition a classification matrix *A* into the minimal number of *ℓ* adjacent sub-matrices *A*^(1)^,…,*A*^(*ℓ*)^ having 6 rows and *m*_1_,…,*m*_*ℓ*_ columns such that $A_{j,k_{1}}^{\left(i\right)}=A_{j,k_{2}}^{\left(i\right)}$ for *j* in 1, …, 6 and *k*_1_, *k*_2_ in 1,…,*m*_*i*_; this is to say, our partition of *A* ensures all columns in submatrix *A*^(*i*)^ are identical and that two consecutive submatrices differ (Fig A in S1 Illustrated methods). Clearly, the first column of any submatrix also characterizes the whole submatrix. We ignore sub-matrices of less than 50 columns in length and refer to the sequence of the first columns of the remaining submatrices as the sequence of *n* pre-patterns $P=\left(A_{\left(1\cdots 6,1\right)}^{\left(i\right)}:\text{ncols}\left(A^{\left(i\right)}\right)\geq 50\right)=P^{\left(1\right)},P^{\left(2\right)},\ldots ,P^{\left(n\right)}$. We form these pre-patterns (Fig B in S1 Illustrated methods) for the 7,224 six-way alignments in the EPO dataset mentioned above.

Our ultimate goal was to place patterns describing the status of ERVs located at orthologous positions in their host genomes at the tips of a phylogenetic tree. To achieve this objective the sequences of pre-patterns described above were parsed into bona-fide site patterns characterizing the state of orthologous ERV loci in each species. The first stage of this parsing process utilized a modified version of Thompson’s Construction Algorithm [32] to identify all contiguous subsequences of pre-patterns of length 3 or more having the following properties: (i) anchored at both ends by a pre-pattern of unannotated nucleotides (all-ds); (ii) containing a common solo-LTR or full-length ERV in the same row of every pre-pattern between the aforementioned anchors; and (iii) containing an entry coded as a gap in the same row of every pre-pattern between the aforementioned anchors (Fig C in S1 Illustrated methods). More precisely, using $P_{q}^{\left(i\right)}$ to denote the *q*th element of the *i*th vector in *P*, we identify the set *S* containing all subsequences of pre-patterns *P*^(*i*)^, *P*^(*i*+1)^, …, *P*^(*j*)^ of length *j* − *i* + 1 ≥ 3 such that: (i) *P*^(*i*)^ = *P*^(*j*)^ = (d, d, d, d, d, d)^⊤^; (ii) there exists *q* such that $P_{q}^{\left(k\right)}=P_{q}^{\left(k+1\right)}=s-x-y$ or $P_{q}^{\left(k\right)}=P_{q}^{\left(k+1\right)}=c-x-y$ is satisfied for all *k*: *i* < *k* < *j* − 1; and (iii) there exists *q* such that $P_{q}^{\left(k\right)}$ is gapped for all *k*: *i* < *k* < *j*. These conditions are sufficient to identify contiguous subsequences that can be interpreted as site patterns.

The set *S* of subsequences of pre-patterns that we have identified so far are unambiguously gapped in one species and unambiguously contain an ERV in one species. One example of such a subsequence is the following:

$$S^{\left(\cdot \right)}={\left(d,d,d,d,d,d\right)}^{⊤},{\left(s,s,s,c,g,g\right)}^{⊤},{\left(d,d,d,d,d,d\right)}^{⊤}.$$

As we always write our pre-patterns in the same order (human, chimp, gorilla, orangutan, macaque, and marmoset) one can see that this example pattern describes an ERV that is missing in macaque and marmoset (the 5th and 6th elements), is present as a solo-LTR in human, chimp, and gorilla (the 1st–3rd positions), and is present as a partial or full-length ERV in orangutan (the 4th position). In fact, we are interested coding patterns that are gapped in marmoset (our outgroup) and present in at least one other species. We code our site patterns using the following notation: an absent sequence is coded as 0; a solo-LTR is coded as x (the letter x being reminiscent of both deletion and recombination); and an ERV present in either partial or full-length form is coded as a 1. It is clear that in our example we would want to assign the site pattern (x, x, x, 1, 0, 0)^⊤^ to our subsequence. Indeed, it is clear that for any subsequence in *S*, we can reasonably assign a 1 or an x as appropriate to the row fulfilling condition (ii) and also assign a g to the row fulfilling condition (iii). Nevertheless, in general, we have constructed *S* requiring unambiguity in only two of six positions. To assign the correct coding to the remaining four positions we applied the following heuristic to subsequences in *S*.

#### Converting pre-patterns to site patterns

Consider the subsequence of pre-patterns *S*^(⋅)^ = *P*^(*i*)^, …, *P*^(*j*)^. We wish to form site pattern *U*^(⋅)^ = (*u*_1_, …, *u*_6_)^⊤^. We know by construction that *u*_6_ = g (absence in marmoset) and that *u*_*q*_ = 1 or *u*_*q*_ = x for some *q* (evidence of ERV insertion in some species). We wish to assign *u*_*q*′_ for *q*′ ≠ *q* in 1, …, 5. To do this we apply the following steps to each subsequence in *S*:

- **if** $P_{q^{\prime }}^{\left(k\right)}=s$ for some *i* < *k* < *j* **then** set *u*_*q*′_ ← x;
- **if** $P_{q^{\prime }}^{\left(k\right)}=c$ for some *i* < *k* < *j* **then** set *u*_*q*′_ ← 1;
- **if** $P_{q^{\prime }}^{\left(k\right)}=d$ for all *i* < *k* < *j* **then**
  {**if** we have already assigned a 1 in the previous steps **then** set *u*_*q*′_ ← 1
**else** set *u*_*q*′_ ← x};
- if $P_{q^{\prime }}^{\left(k\right)}=g$ for all *i* < *k* < *j* then set *u*_*q*′_ ← 0.

The above methods convert pre-patterns to site patterns by combining homology and processed RepeatMasker annotations but do not take any account of the length of sequences. Each pattern assigns an integration state of 0 (absence), 1 (presence) or x (presence in solo-LTR form) to orthologous ERV loci in six species. In a final post-processing phase we make use of the underlying alignment to remove any patterns in which (i) the 5’ flank *P*^(*i*)^ of a pattern is backed by less than 100 nucleotides, (ii) the 3’ flank *P*^(*j*)^ of a pattern is backed by less than 100 nucleotides, or (iii) the inter-flank region *P*^(*i*+1)^, …, *P*^(*j*−1)^ of a pattern is backed by less than 250 nucleotides. In addition, where patterns are mixed, that is patterns contain both 1s and xs, we apply a procedure that converts any particular x to a 1 if 85% of the called sequence backing the interflanking region of a position annotated as x is identical to called sequence in any of the corresponding aligned sequence backing a position annotated as 1. The rationale for this action is that our alignment allows us to apply reasoning by homology that is not available to RepeatMasker or REannotate that operate on single sequences only. Every mixed pattern was examined by hand to ensure that such reasoning was appropriate. We then used these patterns (Fig D in S1 Illustrated methods) as an input to our phylogenetic model that is described in the following section.

### Phylogenetic insertion and deletion model

Consider the primate phylogeny *T* reproduced in Fig 1 where the branch length immediately below node *i* is denoted *T*_*i*_ and takes the value given by [11]. We wish to relate subsets of our sampled site patterns *U* (described above), for example, the subset of patterns relating to HERV-H, to an insertion process on the tree *T* as well as to one of two potential deletion processes, also on *T*, between which we wish to discriminate. The insertion process is assumed to be Poisson and the deletion process is assumed to be either Weibull or exponential. The exponential deletion model is a nested submodel of the Weibull deletion model. We first discuss insertion and then discuss deletion.

#### Insertion

A site in a genome can be assigned one of three states at any particular time: absent or 0 (a pre-integration site); insertion or 1 (contains an ERV); and deleted or x (contains a solo-LTR). Over time, a site may transition from the absent state to the insertion state to the deleted state. That is, only the following state transitions can occur: 0 → 1 → x. Five of the branches of *T* are external and their tips relate to observations *U*. Because we use marmoset as an outgroup, in our patterns *u*_6_ is always equal to 0 and we will ignore it from now on. Given the permitted state transitions, it is clear the first five positions of a pattern unambiguously identify the branch of *T* on which an ERV first appeared. Beyond this the patterns provide no further temporal information. We consider the insertion process on any given branch *i* of *T* to be a Poisson process that is fully described by rate parameter Φ_*i*_. If, for a dataset under analysis, there are *N*_*i*_ insertions on branch *i*, then writing *ϕ*_*i*_ = Φ_*i*_ *T*_*i*_, the likelihood of all insertions on *T* is given by

$$\prod_{i=1}^{9}\frac{e^{-\phi_{i}}\phi_{i}^{N_{i}}}{N_{i}!}.$$

#### Deletion

**Fig 4:**
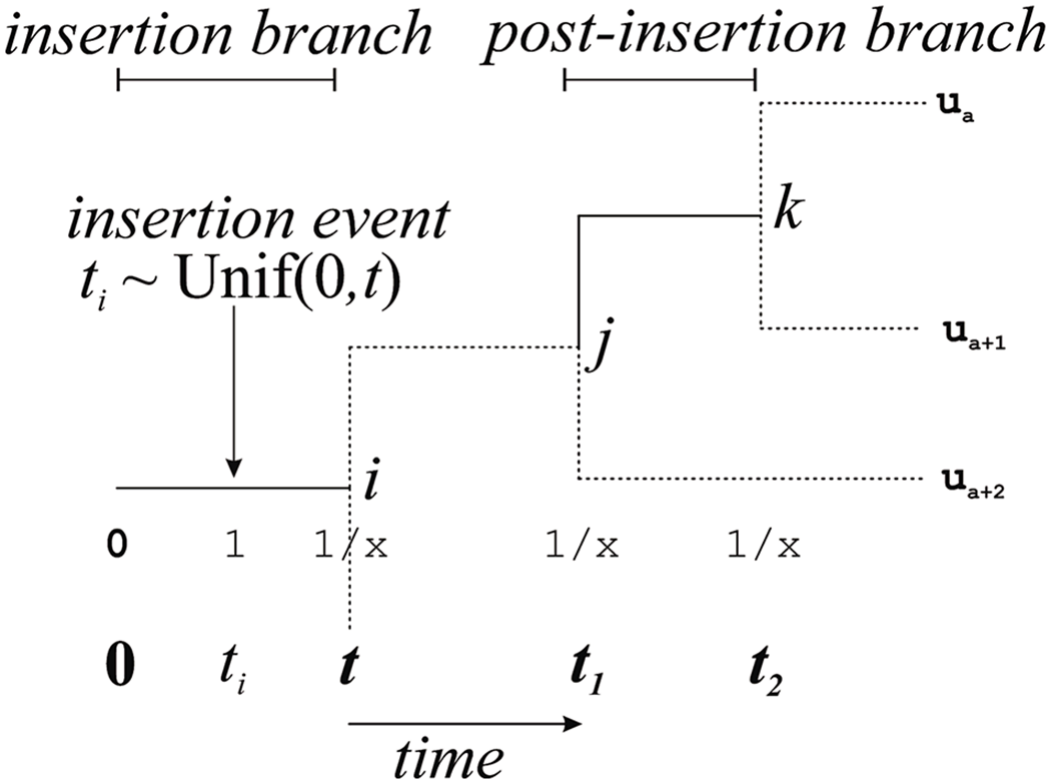
The phylogenetic abstraction on which we describe insertion and deletion processes. For a given site pattern an insertion branch is uniquely determined. In this hypothetical example an insertion occurs on branch *i* at time *t*_*i*_, somewhere between time 0 and *t*. Our deletion model considers all possible state transitions (1 → 1 or 1 → x) on post-insertion branches, internal (here *j* and *k*) or otherwise (observable via site patterns and here labelled with subscripted us).

After entering the insertion state 1, a site may transition to the deleted state x. A site pattern provides an observation of the final state of a site in any particular linage but it will not necessarily describe the state of a site at internal nodes. For example, the site pattern (x, 0, 0, 0, 0)^⊤^ implies that the state at the corresponding site was 0 at all internal nodes. On the other hand, the pattern (x, x, 0, 0, 0)^⊤^ implies that the state at the corresponding site was 0 at all internal nodes except for hc, at which a state of 1 or x are both consistent with the evidence: in the former case a deletion (transition from 1 to x) occurred independently on the branches h and c, while in the latter case a single deletion occurred on the branch hc prior to the human-chimp split. We use a deletion process to consider the probability of transition from 1 to x under all possible assignments of states to internal nodes (Fig 4 and Fig E in S1 Illustrated methods).

To describe our deletion model we need to consider two kinds of branch, the insertion branch and the post-insertion branch. Fig 4 provides an abstract visualization of this concept. For any particular pattern, the insertion branch is uniquely identified as all nodes beneath the insertion branch have state 0 and all nodes above the insertion branch have state 1 or state x. The corresponding post-insertion branches are all those branches above the insertion branch in the tree.

#### Exponential deletion model

We will first consider the deletion process under an exponential model whereby deletion occurs with a constant probability over time. The exponential decay process is assumed common to all branches and parameterized by rate parameter *ψ*. Consider an insertion at time *t*_*i*_ on the branch leading to node *j*. Let *t*_0_ = 0 be the time at the origin of the branch leading to node *j* and time *t* = *T*_*j*_ be the termination of the branch. Under an exponential deletion model the probability of deletion given an insertion at *t*_*i*_ is simply Pr(0 → x|*t*_*i*_) = 1 − *e*^−*ψ*(*t*−*t*_*i*_)^. As insertion follows a Poisson process the time of insertion *t*_*i*_ of any particular ERV on the branch will be uniformly distributed between *t*_0_ and *t*. Therefore, taking advantage of the fact that expectation $E\left[g\left(X\right)\right]=\int_{-\infty }^{\infty }g\left(x\right)f_{x}\left(x\right)\;dx$ for random variable *X* with density function *f*_*x*_ and g:R→R, the probability of changing from state 0 to state x during interval 0 to *t* (see Fig 4 and Fig F in S1 Illustrated methods) is

$$\mathrm{Pr}\left(0\to x\right)=\int_{0}^{t}\frac{1}{t}\left[1-e^{-\psi (t-t_{i})}\right]\;dt_{i}=1+\frac{e^{-\psi t}-1}{\psi t},$$

while the probability that an ERV is not deleted on the insertion branch is Pr(0 → 1) = 1 − Pr(0 → x).

On a post-insertion branch the situation is much simpler as the memoryless property of an exponential decay process ensures that we do not need to consider the time during which a site has been in a particular state when calculating whether it will change state over any subsequent period. Consider the post-insertion branch from node *j* to node *k* of length *t*_2_ − *t*_1_ (Fig 4 and Fig G in S1 Illustrated methods). If the site is in state 1 at node *j* then the probability of no state change is Pr(1 → 1) = *e*^−*ψ*(*t*_2_−*t*_1_)^ and the probability of a deletion is Pr(1 → x) = 1 − *e*^−*ψ*(*t*_2_−*t*_1_)^. As the state x is absorbing, Pr(x → x) = 1, while all other transitions on post-insertion branches have zero probability.

#### Weibull deletion model

We now consider a Weibull process, under which the probability of a deletion occurring during any small time interval may increase or decrease given the age of an insertion. The Weibull process is again assumed common to all branches, but requires two parameters: a rate parameter *ψ* and a shape parameter *ω*. For an insertion branch, the appropriate probability of seeing two state transitions (Fig 4 and Fig H in S1 Illustrated methods) is

$$\begin{array}{ll} \text{Pr}(0\to x) & =\int_{0}^{t}\frac{1}{t}\left[1-e^{-{\left[\frac{t-t_{i}}{\psi }\right]}^{\omega }}\right]\text{d}t_{i}\\ & =1+\frac{\psi \left[\Gamma \left(\frac{1}{\omega },{\left(\frac{t}{\psi }\right)}^{\omega }\right)-\Gamma \left(\frac{1}{\omega }\right)\right]}{\omega t}, \end{array}$$

where $\Gamma \left(z\right)=\int_{0}^{\infty }t^{z-1}e^{-t}\;dt$ and where $\Gamma \left(\alpha ,z\right)=\int_{z}^{\infty }t^{\alpha -1}e^{-t}\;dt$. The probability that an ERV is not deleted on the insertion branch remains the only other possibility so that Pr(0 → 1) = 1 − Pr(0 → x).

The essential feature of a Weibull process is that it can describe deletion rates that vary given the age of an ERV. This means that under a Weibull model the probability of a state change from 1 to x on the post-insertion branch from node *j* to node *k* is no longer independent of insertion time *t*_*i*_. Consider again the possibility of a state change on a branch of length *t*_2_ − *t*_1_ (Fig 4 and Fig I in S1 Illustrated methods). Under a Weibull process the probability of deletion takes into account uncertainty over insertion time *t*_*i*_ giving

$$\mathrm{Pr}\left(1\to 1|t_{i}\right)=\int_{0}^{t}\frac{1}{t}\left\{\frac{e^{-{\left[\frac{t_{2}-t_{i}}{\psi }\right]}^{\omega }}}{e^{-{\left[\frac{t_{1}-t_{i}}{\psi }\right]}^{\omega }}}\right\}\;dt_{i}$$

and Pr(1 → x|*t*_*i*_) = 1 − Pr(1 → 1|*t*_*i*_). These values can be numerically evaluated for all branches and distances on our tree *T*. As before, Pr(x → x) = 1 while all other transitions on post-insertion branches are impossible.

### Combining site patterns and the phylogenetic model for maximum likelihood estimation

We have now completely specified the state transition probabilities for individual branches under a strict exponential model and under a Weibull model. To compute the likelihood of a site pattern given a tree and a deletion model we use a dynamic programming algorithm that is similar to Felsenstein’s pruning algorithm [33]. In the case that we use a Weibull deletion model, the algorithm must be modified to keep track of the insertion branch when considering the probability of transitions on post-insertion branches.

The above description showed how to calculate the probability of insertions as well as the probability of all post-insertional state transitions that may occur. We have also described how to sample site patterns from primate genomes. Therefore we are ready to describe how to calculate the probability of a set of site patterns *U* given a tree *T*, an insertion model *M*_*i*_ and a deletion model *M*_*d*_. For each of *n* site patterns *U*^(*j*)^ we can identify the insertion branch for that pattern, and hence the subtree *T*^(*j*)^ of *T* that includes only the insertion branch and its descendants i.e. any post-insertion branches. To calculate the likelihood of the site patterns we compute:

$$\mathrm{Pr}\left(U|M_{i},M_{d}\right)=\prod_{i=1}^{9}\frac{e^{-\phi_{i}}\phi_{i}^{N_{i}}}{N_{i}!}\prod_{j=1}^{n}\mathrm{Pr}\left(U^{\left(j\right)}|M_{d},T^{\left(j\right)}\right),$$

where the first product gives the likelihood of the insertions and the second product uses the aforementioned dynamic programming method to sum over all possible post insertion state transitions.

The insertion model *M*_*i*_ has 9 parameters, the 9 insertion rates in Φ. The deletion model has one parameter if it is strictly exponential (the rate parameter *ψ*_*e*_) or two parameters (*ψ*_*w*_ and the additional shape parameter *ω*) in the case that it is Weibull. By repeatedly computing the likelihood of our site patterns we can numerically maximize the logarithm of Pr(*U*|*M*_*i*_,*M*_*d*_) using code written in the MATLAB language. In practice we performed simulated annealing using simulannealbnd (limited to 5 minutes per replicate during bootstrapping [34] i.e. when sampling the site patterns with replacement) followed by gradient descent using fmincon. As the insertion process is independent of the deletion process we were able to carefully verify our maximum likelihood results using grid search and gradient descent from random starting points.

## Supporting Information

S1 Tabular data A Microsoft Excel spreadsheet file containing site patterns (including their original locations in the genome) as well as details of the viral library used to check the RepeatMasker classification of ERVs. (XLSX)

S1 Illustrated methods A PDF file that contains the supplementary figures A–I that complement the text in the methods section of the main manuscript. (PDF)
