# Supplementary material for: Phylogenetic Analysis Reveals That ERVs "Die Young" but HERV-H Is Unusually Conserved
Source: PLoS Comput Biol. 2016 Jun 13;12(6):e1004964. doi: 10.1371/journal.pcbi.1004964 (PMC4905674; doi:10.1371/journal.pcbi.1004964)
Supplement: S1 Illustrated methods — (PDF) [file pcbi.1004964.s002.pdf]

S2 Illustrated methods

This document contains additional figures that illustrate the processes and models described in the Methods section of the main manuscript.

Sampling site patterns

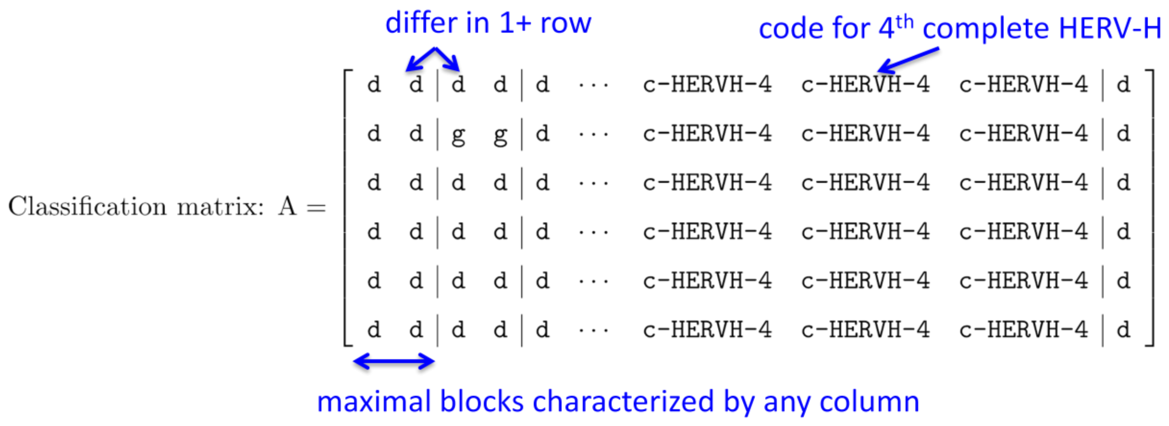

Figure A: Each position in a matrix representation of a six-way EPO alignment can be classified as one of the following: an unannotated nucleotide coded as **d**; an unannotated gap coded as **g**; the *i*th solo-LTR having identifier *id* and coded as **s**-*i*-*id*; or the *i*th partial or full-length ERV having identifier *id* and coded as **c**-*i*-*id*. We partition classification matrix $\boldsymbol{A}$ into l adjacent submatrices so that all columns in submatrix $\boldsymbol{A}^{(i)}$ are identical and so that two consecutive submatrices differ.

$$\text{Prepatterns: } \mathrm{P} = \left(\cdots, \begin{bmatrix} \texttt{d} \\ \texttt{d} \\ \texttt{d} \\ \texttt{g} \\ \texttt{d} \\ \texttt{d} \end{bmatrix}, \begin{bmatrix} \texttt{d} \\ \texttt{d} \\ \texttt{d} \\ \texttt{d} \\ \texttt{d} \\ \texttt{d} \end{bmatrix}, \begin{bmatrix} \texttt{s-HERV-H-2} \\ \texttt{s-HERV-H-2} \\ \texttt{s-HERV-H-2} \\ \texttt{c-HERV-H-2} \\ \texttt{g} \\ \texttt{g} \end{bmatrix}, \begin{bmatrix} \texttt{g} \\ \texttt{g} \\ \texttt{g} \\ \texttt{c-HERV-H-2} \\ \texttt{g} \\ \texttt{g} \end{bmatrix}, \begin{bmatrix} \texttt{d} \\ \texttt{d} \\ \texttt{d} \\ \texttt{d} \\ \texttt{d} \\ \texttt{d} \end{bmatrix}, \begin{bmatrix} \texttt{s-ERV9-1} \\ \texttt{s-ERV9-1} \\ \texttt{s-ERV9-1} \\ \texttt{g} \\ \texttt{g} \\ \texttt{g} \end{bmatrix}, \begin{bmatrix} \texttt{d} \\ \texttt{d} \\ \texttt{d} \\ \texttt{d} \\ \texttt{d} \\ \texttt{d} \end{bmatrix}, \cdots\right)$$

Figure B: The sequence of the first columns of all submatrices of at least 50 columns is referred to as the sequence of pre-patterns $\boldsymbol{P}$.

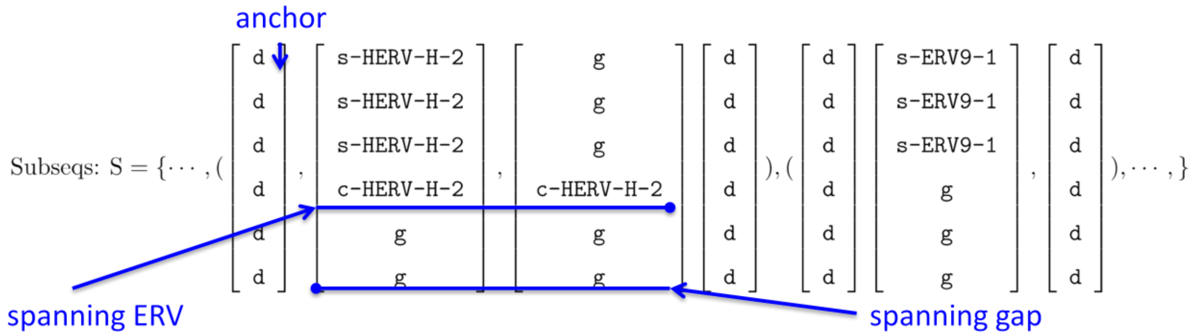

Figure C: To obtain site patterns to place on a phylogeny we parsed the set ***S*** of subsequences of pre-patterns that are (i) anchored by host DNA at either end, that (ii) contain a spanning solo-LTR or full-length ERV, and that (iii) contain a spanning gap.

$$\text{Site patterns: } U = \left\{\cdots, \begin{bmatrix} \mathtt{x} \\ \mathtt{x} \\ \mathtt{x} \\ \mathtt{1} \\ \mathtt{0} \\ \mathtt{0} \end{bmatrix}, \begin{bmatrix} \mathtt{x} \\ \mathtt{x} \\ \mathtt{x} \\ \mathtt{0} \\ \mathtt{0} \\ \mathtt{0} \end{bmatrix}, \cdots, \right\}$$

Figure D: The parsed pre-patterns from ***S*** are converted to site patterns using a heuristic method. These site patterns can then be analysed using a phylogenetic insertion and deletion model.

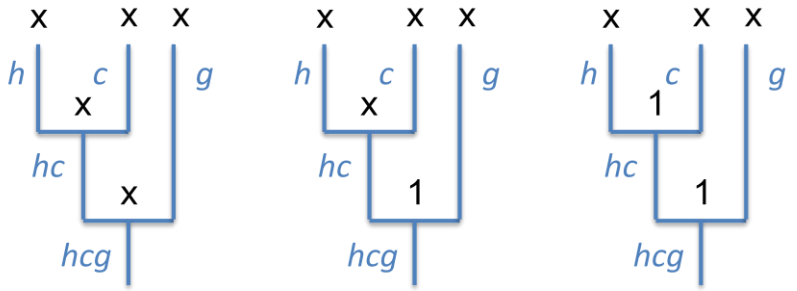

Figure E: Consider an insertion that occurred in a common ancestor of the human, chimpanzee, and gorilla. To calculate the probability of observing solo-LTRs in all present day primates we must consider each of the above three deletion scenarios.

Exponential deletion model

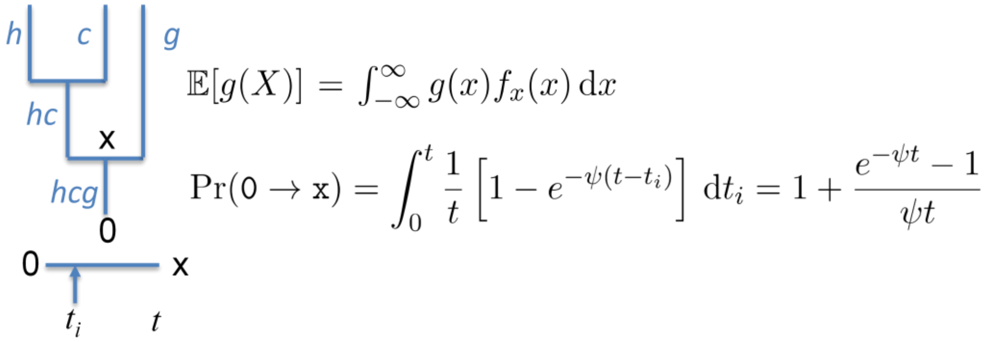

$$\mathbb{E}[g(X)] = \int_{-\infty}^{\infty} g(x) f_x(x)\, \mathrm{d}x$$

$$\Pr(0 \rightarrow \mathrm{x}) = \int_0^t \frac{1}{t} \left[1 - e^{-\psi(t-t_i)}\right] \mathrm{d}t_i = 1 + \frac{e^{-\psi t} - 1}{\psi t}$$

Figure F: Under an exponential model, the probability that an ERV is deleted on an insertion branch has an analytical solution. (The insertion time t_i is uniformly distributed because we assume ERVs arrive according to a Poisson process.)

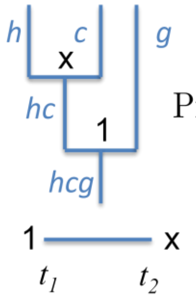

$$\Pr(1 \rightarrow \mathrm{x}) = 1 - e^{-\psi(t_2 - t_1)}$$

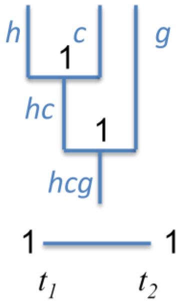

$$\Pr(1 \rightarrow 1) = e^{-\psi(t_2 - t_1)}$$

Figure G: Under an exponential deletion model, on a post insertion branch we need to be able to calculate two kinds of probabilities: the probability that an ERV is deleted (converted to a solo-LTR, left); and the probability that an ERV remains in full-length form (right).

Weibull deletion model

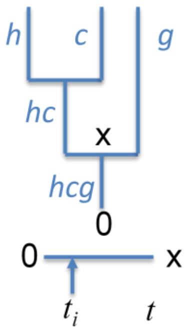

$$\Pr(0 \rightarrow \mathrm{x}) = \int_0^t \frac{1}{t} \left[1 - e^{-\left[\frac{t-t_i}{\psi}\right]^{\omega}}\right] \mathrm{d}t_i = 1 + \frac{\psi \left[\Gamma\left(\frac{1}{\omega}, \left(\frac{t}{\psi}\right)^{\omega}\right) - \Gamma\left(\frac{1}{\omega}\right)\right]}{\omega t}$$

Figure H: Under a Weibull model, the probability that an ERV is deleted on an insertion branch also has an analytical solution. (The insertion time t_i is uniformly distributed because we assume ERVs arrive according to a Poisson process.)

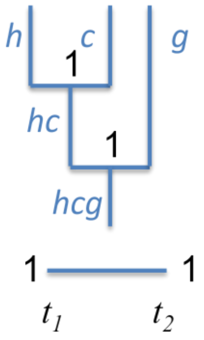

$$\Pr(1 \rightarrow 1|t_i) = \int_0^t \frac{1}{t} \left\{ \frac{e^{-\left[\frac{t_2 - t_i}{\psi}\right]^\omega}}{e^{-\left[\frac{t_1 - t_i}{\psi}\right]^\omega}} \right\} \mathrm{d}t_i$$

Figure I: Under a Weibull model, the probability that an ERV is deleted on a post insertion branch is obtained using the conditional reliability function.
